# Peptide-Protected Gold Nanoclusters Efficiently Ameliorate Acute Contact Dermatitis and Psoriasis via Repressing the TNF-α/NF-κB/IL-17A Axis in Keratinocytes

**DOI:** 10.3390/nano13040662

**Published:** 2023-02-08

**Authors:** Yu Liu, Cong Meng, Yanggege Li, Dongfang Xia, Cao Lu, Jing Lai, Yulu Zhang, Kai Cao, Xueyun Gao, Qing Yuan

**Affiliations:** 1 Center of Excellence for Environmental Safety and Biological Effects, Beijing Key Laboratory for Green Catalysis and Separation, Department of Chemistry, Beijing University of Technology, Beijing 100124, China; 2 College of Chemistry and Material Science, Shandong Agricultural University, Taian 271018, China

**Keywords:** gold nanoclusters, keratinocyte, acute irritant contact dermatitis, psoriasis, NF-κB, IL-17A

## Abstract

Immune-mediated skin diseases have a high prevalence and seriously affect patients’ quality of life. Gold compounds have been considered promising therapeutic agents in dermatology, but the high incidence of adverse reactions have limited their clinical application. There is a great need to develop more effective and less toxic gold-based drugs. Gold nanoclusters fabricated by using peptides (pep-AuNCs) have appeared as potential biomedical nanomaterials because of their excellent biocompatibility, ease of fabrication and unique physicochemical properties. Glutathione (GSH) is an endogenous tripeptide and has been used for lightening the skin color. Therefore, we fabricated a well-defined gold nanocluster with GSH as an example to explore the immunomodulatory effect of AuNCs on a TNF-α-treated human keratinocyte cell line (HaCaT) in vitro, the 12-O-tetradecanoylphorbol-13-acetate (TPA)-induced irritant contact dermatitis (ICD) model and the oxazolone (OXA)-induced psoriatic model in vivo. The results indicated that topically applied AuNCs successfully attenuated the severity of ICD and psoriasis-like lesions. In vitro and in vivo, AuNCs effectively inhibited the abnormal activation of the NF-κB pathway and the consequent overexpression of proinflammatory cytokines in keratinocytes. In particular, the transactivation of IL-17A, the most important cytokine in psoriasis pathology, was effectively inhibited by AuNCs treatment. In addition, AuNCs did not show any obvious cytotoxicity in HaCaT cells at doses even up to 100 µM and did not induce any irritation in the healthy skin and major organs, which indicated their favorable biosafety. These results indicate that biocompatible pep-AuNCs might be a promising gold-based nanomedicine for the treatment of inflammatory skin diseases.

## 1. Introduction

Skin is a crucial physical barrier against exogenous pathogens, acting by preserving an abundance of immune cells and inflammatory mediators [1,2]. Its dysfunction will result in several disorders, including acute irritant contact dermatitis (ICD), atopic dermatitis (AD), psoriasis and others [2,3]. These skin diseases seriously affect patients’ quality of life and have a high prevalence; for instance, psoriasis affects approximately 2–3% of the global population (more than 60 million) [4,5]. Gold drugs have been considered potential therapeutic agents for psoriasis, but the high incidence of nonnegligible adverse reactions has limited their clinical application [6,7,8]. One of the most frequent side effects of chrysotherapy is dermal toxicity, manifested, especially, as nonspecific dermatitis [9,10,11]. Therefore, more efficacious and less toxic gold-based topical therapy options are needed.

Studies have suggested that the side effects of gold drugs are partly due to the organic ligands or their metabolites [6,9]. Therefore, the use of a natural biomolecular ligand might be an ideal strategy. In recent years, gold nanoclusters with peptide ligands (pep-AuNCs) have appeared as potential biomedical materials because of their excellent biocompatibility (causing little irritation or immune responses in vivo), ease of fabrication and unique physicochemical properties [12,13,14]. The latest studies have demonstrated that, in addition to the excellent physical and chemical properties, pep-AuNCs also exhibit unique intrinsic bioactivities and show good potential in the field of disease diagnosis and treatment [15,16,17,18,19,20,21]. AuNCs have been reported to prevent and treat skin infections due to their antimicrobial activity [22,23]. However, their immunomodulatory properties and therapeutic potential in inflammatory skin diseases, such as contact dermatitis and psoriasis, are still unclear.

Accumulating evidence has indicated that cutaneous inflammation initiated from epidermal keratinocytes plays a critical role in the pathogenesis of several skin disorders and is a common therapeutic target [24,25,26]. Keratinocytes, which make up 90% of the epidermal cells in the outermost layer of the skin, are susceptible to proinflammatory stimuli and accountable for triggering inflammatory responses [27,28,29]. Inflammatory cytokines secreted by immune cells, such as tumor necrosis factor-alpha (TNF-α) and interleukin-1 (IL-1), activate keratinocytes and reprogram their differentiation and proliferation, resulting in increased epidermal thickness and chemokine production to bring more immune cells to the injury site [29,30,31]. This positive feedback cycle between immune cells and keratinocytes leads to severe skin inflammation [32].

Nuclear factor-κB (NF-κB) is a critical regulator of keratinocyte differentiation and proliferation. Previous studies have demonstrated that this signaling molecule is involved in the pathogenesis of psoriasis by upregulating the production of chemokines and pro-inflammatory cytokines, such as IL-6, TNF-α and IL-17A [33,34,35,36,37]. In psoriasis, the key target of TNF-α is the NF-κB signaling pathway. [36]. Members of the NF-κB family, including NF-κB1 (p50/p105), NF-κB2 (p52/p100), p65 (RelA), RelB and c-Rel, either homodimerized or heterodimerized, travel from the cytoplasm to the nucleus after activation and work as transcription factors to induce the transcription of inflammatory cytokines and chemokines [38]. Notably, NF-κB could bind to the promoter of IL-17A and transactivate it in HaCaT cells, indicating a critical role of NF-κB in psoriasis. Thus, the suppression of NF-κB in keratinocytes might be advantageous for psoriasis treatment [31,39].

Glutathione (GSH) is an endogenous thiol tripeptide and has been commonly used to lighten skin color topically, which suggests its efficient percutaneous absorption and skin compatibility [40,41]. In addition, a comprehensive study indicated that GSH-protected AuNCs possess better in vivo metabolic characteristics (can be efficiently eliminated through the kidneys) and higher biosafety (causing little obvious toxic effects or damage to the main organs in vivo) than bovine serum albumin (BSA)-protected AuNCs, thus showing greater potential in biomedical applications [42]. Therefore, in this study, we prepared well-established pep-AuNCs with γ-GSH as the ligand and explored their topical therapeutic effects in 12-O-tetradecanoylphorbol-13-acetate (TPA)-induced irritant contact dermatitis and the oxazolone (OXA)-induced psoriatic mice model. In both models, compared to the control mice, the mice administered AuNCs topically displayed significant decreased erythema and skin thickness and reduced infiltration of proinflammatory immune cells. Mechanistic studies using the human skin keratinocytes HaCaT revealed that the AuNCs could reduce the TNF-α-induced upregulation of IL-1β, TNF-α, IL-6 and IL-17A, due to reduced activation of the NF-κB pathway in vitro and in vivo. Analysis of the in vivo distribution of the gold element and histopathological examination of the major organs showed that the AuNCs were mainly excreted by the kidneys and did not cause obvious tissue toxicity to the major organs, showing good biosafety. These results revealed pep-AuNCs as a potential gold-based medication against inflammatory skin diseases and provide novel insights into the medicinal application of AuNCs.

## 2. Materials and Methods

### 2.1. Materials

Glutathione (GSH) was purchased from Sigma-Aldrich (St. Louis, Missouri, USA) (purity: 95%); 12-O-tetradecanoylphorbol-13-acetate (TPA) and oxazolone (OXA) were purchased from Sigma-Aldrich (St. Louis, Missouri, USA). Recombinant human TNF-alpha protein was obtained from Yeasen Biotechnology (Shanghai, China). The human keratinocyte cell line HaCaT was purchased from BeiNa Culture Collection (Xinyang, China). Fetal bovine serum (FBS) and Dulbecco’s modified Eagle medium (DMEM) were acquired from Gibco (USA). The Cell Counting Kit 8 (CCK-8) was purchased from Dojindo Laboratories (Kumamoto, Japan). Antibodies against IKKβ, p-IKKβ, IκBα, p-IκBα, p65, p-p65 and β-actin were purchased from Cell Signaling Technology (Beverly, MA, USA). The antibody against IL-17A was purchased from Beyotime Biotechnology (Shanghai, China). HRP-labeled Goat Anti-Rabbit IgGs (H+L) were obtained from Beyotime Biotechnology (Shanghai, China).

### 2.2. Synthesis of GSH-Protected AuNCs

The synthesis of glutathione-protected gold nanoclusters was modified from our previous reports [43]. We mixed freshly prepared GSH (30 mM, 100 mL) with an HAuNCl_4_·3H_2_O (20 mM, 100 mL) solution and stirred (500 rpm) the mixture at room temperature for 10 min. The temperature of the reaction was raised to 70 °C for 12 h with gentle stirring (500 rpm). After placing at room temperature for another 12 h in the dark, we synthesized strongly orange-emitting pep-AuNCs. For the following investigations, the prepared pep-AuNCs were ultrafiltered (Millipore, MWCO: 3 kDa) to remove the free gold ions and kept at 4 °C.

### 2.3. Characterization of the Au Nanoclusters

A fluorescence spectrophotometer (Shimadzu RF-5301, Kyoto, Japan) was used to acquire the photoluminescence (PL) spectra of Au nanoclusters. The hydrate particle size of the as-prepared AuNC was analyzed by dynamic light scattering (Malvern Zetasizer Nano S, Malvern, UK). The synthetic AuNCs were observed using a High-Resolution Transmission Electron Microscope (HRTEM, HT7700 Exalens) at the accelerated voltage of 200 kV.

### 2.4. Experiments In Vitro

#### 2.4.1. Culture and Treatment of Cells

HaCaT cells were grown in Dulbecco’s Modified Eagle medium (DMEM) supplemented with 10% heat-inactivated fetal bovine serum (FBS) and penicillin/streptomycin (100 µg/mL) at 37 °C in a humidified atmosphere containing 5% carbon dioxide. The cells were used for the experiments when they were in logarithmic growth phase.

#### 2.4.2. Cell Viability Assay with Cell Counting Kit-8

The Cell Counting Kit-8 (CCK-8) was used to evaluate cytotoxicity. HaCaT cells were seeded at a density of 1 × 10^4^ cells per well in 96-well plates. The cells were incubated with different concentrations of Au (5, 10, 20, 50 and 100 µM) for 24 h. The medium was then replaced with a new medium containing 10% (*v*/*v*) CCK-8 and cultured for 1 hour. A SpectraMax M2 microplate reader (Molecular Devices, Sunnyvale, CA, USA) was used to detect the absorbance at 450 nm.

#### 2.4.3. Protein Extraction and Western Blotting

HaCaT cells were seeded at a density of 3 × 10^5^ cells per well in a 6-well plate overnight, then incubated with GA (Au concentrations of 0, 50, 100 µM) or GSH (100 μM) for 24 h before being stimulated with TNF-α (20 ng/mL) for 30 min. The whole proteins were extracted from HaCaT cells with RIPA Lysis Buffer (Beyotime Biotechnology, Shanghai, China). The proteins in equal quantities were divided by 12% SDS-PAGE and then transferred to PVDF membranes. After blocking at room temperature for 1 hour, the membranes were incubated overnight at 4 °C with specific antibodies, followed by a 1-hour incubation with secondary antibodies at room temperature. The immunoreactive bands were visualized by using the enhanced chemiluminescence (ECL) reagent (GE Healthcare, London, UK), and the optical density was evaluated using ImageJ software (NIH, Bethesda, MD, USA).

#### 2.4.4. RNA Extraction and Real-Time Quantitative PCR (qPCR)

HaCaT cells were cultured in a medium containing 20 ng/mL of TNF-α and various doses of GA (the Au concentrations were 0, 50 and 100 µM) or GSH (100 µM) for 24 h in a 12-well plate. According to the manufacturer’s instructions, total RNA was isolated from HaCaT cells using an extraction kit (RNAeasy™ Animal RNA Isolation Kit with Spin Columnthe, Beyotime, Beijing, China). The cDNA was produced in a 20 μL reaction system using a reverse-transcription reagent (TransScript^®^ Uni All-in-One First-Strand cDNA Synthesis SuperMix for qPCR, TransGen Biotech, Beijing, China). The LightCycler^®^ 96 instrument (Roche, Basel, Switzerland) and a qPCR kit (PerfectStart^®^ Green qPCR SuperMix, TransGen Biotech, Beijing, China) were used to conduct the real-time quantitative PCR. The relative expression of IL-6, TNF-α, IL-1β and IL-17A mRNA was standardized to the quantity of GAPDH in the same samples using a relative quantification approach (2^-ΔΔCt^ method). We used the following primers: for TNF-α, forward 5′-AGC CCA TGT TGT AGC AAA CC-3′ and reverse 5′-GGC ACC ACC AAC TGG TTA TC-3′; for IL-6, forward 5′-AAA GAG GCA CTG GCA GAA AA-3′ and reverse 5′-TTT CAC CAG GCA AGT CTC CT-3′; for IL-1β, forward 5′-AGG CCT CTC TCA CCT CTC CT-3′ and reverse 5′-AGA ATG TGG GAG CGA ATG AC-3′; for IL-17A, forward 5′-TCC CAC GAA ATC CAG GAT GC-3′ and reverse 5′-GGA TGT TCA GGT TGA CCA TC AC-3′; for GAPDH, forward 5′-ACT TTG GTA TCG TGG AAG GAC-3′and reverse 5′ -AGT AGA GGC AGG GAT GAT GTT-3′.

### 2.5. Experiments In Vivo

#### 2.5.1. Animals

In the experiments, 8-week-old female C57BL/6J mice were employed. The animals were kept at 19–25 °C with 12 h day/night cycles, with adequate water and mouse diet. All animal procedures were conducted in compliance with the Ethics Committee of Beijing University of Technology, China (Approval No. HS202109001), and carried out in accordance with the National Law on the Use of Experimental Animals (China).

#### 2.5.2. TPA-Induced Ear Inflammation in the Mice

We conducted the experiment with a minor modification from a previously reported approach. The model involved only a single application of 10 μL of 0.03% (*w*/*v*: in acetone) TPA to the outer and inner surfaces of both ears in the mice. The right ear was treated with GA (Au concentration of 50 μM) for 45 min and 2 hours after the TPA application, whereas the left ear was left untreated as a control. An additional set of normal mice was used as normal controls. Ear thickness and weight were assessed 6 h after TPA administration. The animals were then euthanized by cervical dislocation, and tissue samples were collected for further analysis.

#### 2.5.3. OXA-Induced Psoriasis-like Mouse Model

Modifications were made to the previously published method for the OXA-induced model. Briefly, we shaved the fur from the abdomen of each animal the day before the experiment, making sure there were no scratches on the skin. On day −7, the mice were sensitized by applying 100 μL of 1.5% (*w*/*v*: in acetone) OXA to the shaved abdomen. From day −3 to day 1, the mice ears were treated with cream (Cetaphil, Canada) alone or cream + GA once daily. On day 0, the mice ear skin was again stimulated with 0.5% OXA (20 μL per ear) 3 h before treatment with a different cream formulation. Ear thickness was measured 24 h after OXA application, and samples were taken for subsequent experiments.

#### 2.5.4. In Vivo Distribution

Au distribution in the main organs (heart, liver, spleen, lung and kidney) was examined by Inductively Coupled Plasma Mass Spectrometry (ICP-MS) after GA topical treatment (5 mg/kg Au) on the back of BALB/c nude mice once a day, continuously for 7 days. The organ samples were lyophilized, weighed, and predigested with H_2_O_2_ and HNO_3_ (1:3, *v*/*v*) overnight. The predigested samples were digested again using aqua fortis by gentle heating. The remaining 0.1–0.2 mL sample solution was then diluted with 1% HCl and 2% HNO_3_ to a final volume. The sample solutions were analyzed for Au content using ICP-MS (Thermo Elemental X7, Waltham, USA).

#### 2.5.5. Histopathological Examination

The ears tissues were collected and fixed in 4% paraformaldehyde. The dehydrated samples were paraffin-embedded, sectioned (5 µm) and stained with hematoxylin and eosin (H&E) as described in previous studies. At the magnification of 40x, a representative region was chosen for the qualitative light microscopic investigation of the inflammatory cellular response. Inflammatory infiltration and tissue damage in the ear tissue were quantitatively analyzed by a professional investigator. Then, a pathological score was obtained based on the inflammation grade.

#### 2.5.6. Immunohistochemical Staining

For immunohistochemistry staining, paraffin-embedded tissue slices were deparaffinized twice (5 min each) with xylene according to a previously reported modified method. After washing with 100% ethyl alcohol, the slides were moved to 95% ethyl alcohol for 3 min before being treated for 15 min with 3% hydrogen peroxide in distilled water to inhibit endogenous peroxidase activity. The slides were then washed in PBS, kept in 1% BSA to decrease non-specific staining and incubated overnight at 4 °C with a specific antibody against IL-17A (1:3500). Following the incubation, the slides were washed three times with PBS and treated for 20 min with a secondary antibody. After that, the slides were incubated with a DAB substrate solution for 15 min, then washed in water and counterstained for 30–60 s with hematoxylin before being dried, fixed and examined under a microscope.

#### 2.5.7. Immunofluorescence Staining

Paraffin-embedded slides were dewaxed in xylene for 8 min (three times) and permeated in 100%, 90%, 80% and 60% alcohol for 8 min, as previously reported. After that, the slides were subjected to a 15min treatment at room temperature with 3% hydrogen peroxide. The slides were rinsed three times with PBS for 5 min. The slices were incubated with goat serum at 37 °C for 30 min. After blocking, the slides were incubated with primary antibodies specific for phosphorylated p65 (1:50), IL-6 (1:50) and TNF-α (1:250). The next day, the slides were rinsed with PBS again and incubated at 37 °C for 20 min with a secondary antibody (Cy3-labeled goat anti-rabbit 1:100). Before being examined under a fluorescence microscope, the slides were counterstained with DAPI (1 µg/mL) for 30 min after being washed three times with PBS.

### 2.6. Statistical Analyses

All experimental results in this paper are presented as mean ± standard deviation (SD). The experimental group (intervention group) was compared to the control group using one-way analysis of variance (one-way ANOVA), and *p* < 0.05 was judged statistically significant. The software used for all data processing was SPSS software version 19.0 (Chicago, IL, USA).

## 3. Results and Discussions

### 3.1. Characterization of GSH-Protected Gold Nanoclusters

**Figure 1:**
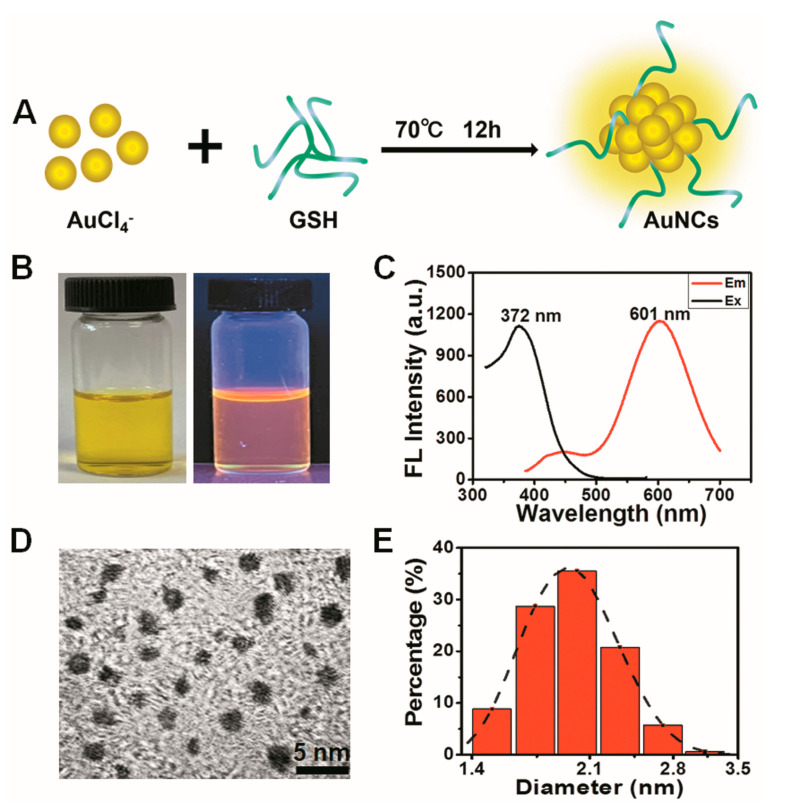
Characterization of GSH-protected gold nanoclusters. (**A**) Schematic of the synthesis of GA. (**B**) Visible (left) and ultraviolet (right) pictures of the GA solution. (**C**) Fluorescence excitation (375 nm) and emission spectra (601 nm) of GA. (**D**) Image of GA captured with a high-resolution transmission electron microscope (HRTEM). (**E**) Hydrated particle size distribution of the synthesized Au nanoclusters.

GSH-protected gold nanoclusters (AuNCs) with high biocompatibility were prepared by a one-pot method according to our previously reported procedure and were designated GA (Figure 1A) [43]. The as-prepared AuNCs are light yellow in solution under visible light, while showing an orange red photoluminescence under UV light (Figure 1B). The photoluminescence excitation and emission peaks of GA are located at 372 nm and 601 nm, respectively, which shows a large Stokes shift for about 230 nm (Figure 1C). High-resolution transmission electron microscope (HRTEM) images revealed that the AuNCs were well dispersed in water and had an ultrasmall size (Figure 1D). The average hydrodynamic size of GA, as established by dynamic light scattering (DLS) detection, was around 2.04 nm (Figure 1E). These characteristic properties are in agreement with our prior studies, indicating a stable preparation of GA [43].

### 3.2. GA Suppress the TNF-α-Stimulated NF-κB Pathway and the Subsequent Upregulation of Proinflammatory Cytokines in Human Keratinocytes

**Figure 2:**
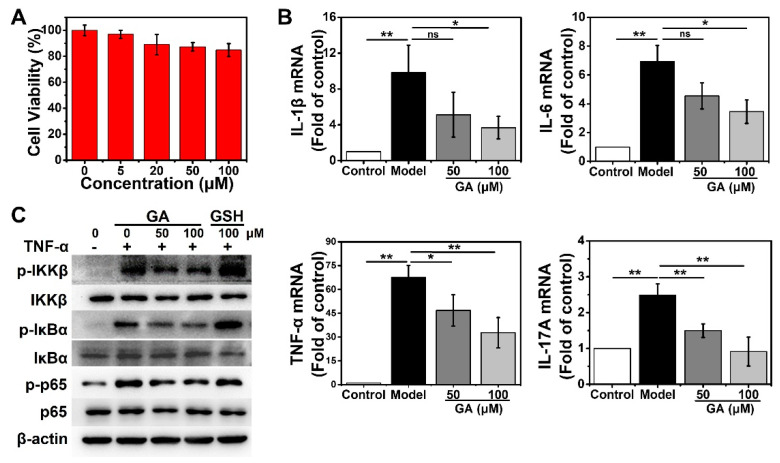
Effect of Au nanocluster on NF-κB pathway activation and proinflammatory cytokines expression in human keratinocytes. (**A**) CCK8 assay for cell viability of HaCaT cells after incubation with the indicated concentrations of GA for 24 h. The data are presented as mean ± SD of triplicate experiments. (**B**) Effects of GA on TNF-α (20 ng/mL)-induced transcription of IL-1β, IL-6, TNF-α and IL-17A in HaCaT cells after 24 h of incubation detected by RT-qPCR. The data are provided as mean ± SD of triplicate experiments; * *p* < 0.05, ** *p* < 0.01 and n.s., not significant. (**C**) Western blotting was used to determine the expression and phosphorylation of IKKβ, IκBα and p65 following a 24 h incubation with TNF-α and GA or free glutathione. The loading control was β-actin. Presented is one result from three distinct trials.

To evaluate the therapeutic potential of GA on inflammatory skin diseases in vitro, a normal human keratinocyte cell line, HaCaT, was used as a model. The cytotoxicity of GA to HaCaT cells was examined using the CCK8. After incubating with different doses of GA (0, 5, 20, 50, 100 μM) for 24 h, HaCaT cells did not show any obvious decline in viability, even at the dose of Au up to 100 μM (Figure 2A). Clinical and genetic studies have highlighted the pivotal roles of several inflammatory cytokines derived from keratinocytes during the pathogenesis of inflammatory skin diseases, including IL-1β, IL-6, TNF-α and IL-17A [31,44]. Especially, TNF-α secreted by immune cells can induce the expression of TNF-α and other cytokines by keratinocytes [31]. Considering this, we next used GA to evaluate its anti-inflammatory activity in TNF-α-stimulated HaCaT cells. The RT-qPCR results indicated that TNF-α stimulation induced the overexpression of proinflammatory cytokines, such as IL-1β, IL-6, TNF-α and IL-17A, in HaCaT cells (Figure 2B). However, GA treatment significantly suppressed the transcription of these cytokines in a dose-dependent way (Figure 2B).

NF-κB plays a key role in maintaining the homeostasis of epidermal structure and function [34]. NF-κB signaling is a potential mechanism by which TNF-α promotes the expression of inflammatory factors in keratinocytes to induce skin inflammation [36]. Especially, nuclear NF-κB p65 can bind to the promoter of IL-17A and trigger the overexpression of IL-17A, resulting in spontaneous psoriasis-like skin inflammation [31]. To delineate the underlying mechanism by which GA suppresses TNF-α-induced inflammatory cytokine expression, we examined the status of NF-κB signal in HaCaT cells after GA treatment. The cells were stimulated with TNF-α and treated with GA (50, 100 μM) for 24 h. Western blotting showed that GA could effectively inhibit TNF-α-induced IKKβ activation, IκBα degradation and p65 phosphorylation in a dose-dependent manner (Figure 2C). In contrast, equal doses of free GSH had no such activity, which indicated the inhibitory effect was attributable to the AuNCs (Figure 2C). These results indicated that AuNCs can effectively inhibit keratinocyte activation by suppressing the NF-κB pathway and inflammatory cytokine production.

### 3.3. GA Ameliorate 12-O-Tetradecanoyl Phorbol-13-Acetate (TPA)-Induced Acute Irritant Contact Dermatitis (ICD) In Vivo

**Figure 3:**
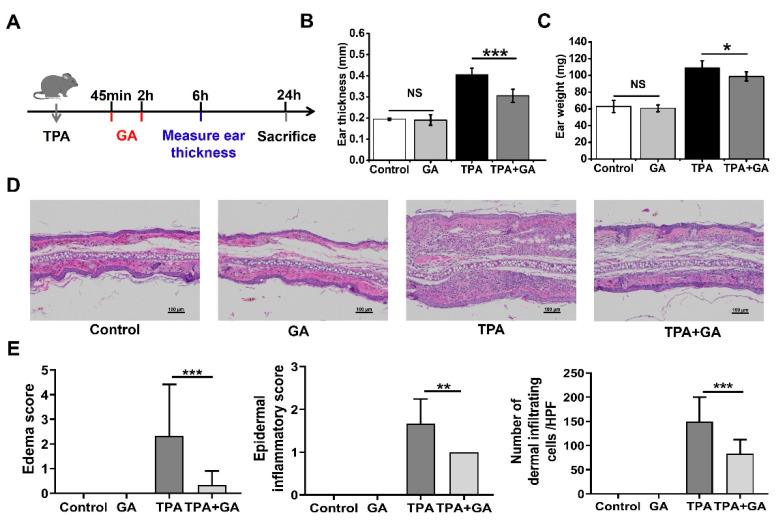
Effect of Au nanoclusters in 12-O-tetrachenol-13-acetate (TPA)-induced acute irritant contact dermatitis (ICD). (**A**) The mice were separated into four groups, and the ICD mouse model was established by applying TPA topically. The thickness (**B**) and weight of the ear (**C**) was measured 6 h after TPA treatment; n = 5 mice per group in the ICD model; * *p* < 0.05, *** *p* < 0.001; n.s., not significant. (**D**) Representative hematoxylin and eosin (H&E)-stained histopathologic slices of mouse ear tissue from each group. The magnification for all images is the same. Scale bar = 100 μm. (**E**) Dermal inflammatory score of the histopathological images. Data are the results of scoring three pathological sections per group and are expressed as the mean ± SD (n = 3); ** *p* < 0.01, *** *p* < 0.001.

ICD is one of the most frequent inflammatory skin disorders [44]. TPA can induce NF-κB activation in mice skin, with symptoms similar to those characteristic of human ICD [45]. To assess the therapeutic activity of GA in acute dermatitis in vivo, we established a mouse model of TPA-induced ear inflammation. The schematic diagram of the model is shown in Figure 3A. We measured the thickness and weight of the ears, which are indicators of ear swelling. After treatment with GA, the increase in ear thickness and weight stimulated by TPA was significantly alleviated (Figure 3B,C). Histopathological analysis of the ear tissue showed a spongy edema and significant infiltration of inflammatory cells in the dermis after TPA stimulation, which was dramatically attenuated by the topical treatment with GA (Figure 3D). However, the vehicle (cream) alone did not ameliorate the TPA-induced symptoms significantly (Figure S1). These phenotypes were further confirmed by the statistical analysis of the histopathological scoring (Figure 3E). There were no significant differences in the indexes between the GA-alone group and the control group, indicating that the AuNCs were not irritating to skin and thus possess good biocompatibility (Figure 3D,E).

### 3.4. GA Attenuate Skin Inflammation and Keratinocyte Abnormality in Oxazolone (OXA)-Induced Psoriasis-Like Mice

**Figure 4:**
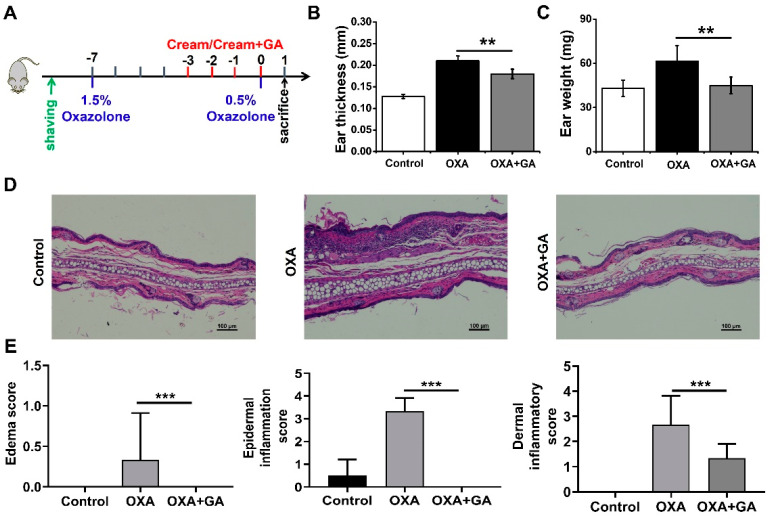
Effect of Au nanoclusters on skin inflammation and keratinocyte abnormalities in oxazolone (OXA)-induced psoriasis. The mice were divided into three groups, n = 5 mice per group. (**A**) depicts a flowchart of the experimental protocol for the psoriasis model. Ear swelling was assessed by measuring ear thickness (**B**) and weight (**C**). Data are presented as the mean ± SD (n = 5); ** *p* < 0.01. (**D**) Images of the H&E staining of the ear of mice in each group treated with OXA or OXA+GA. Scale bar = 100 μm. (**E**) Histological score of inflammatory infiltration and edema in the ears of the mice. Data are presented as the mean ± SD (n = 3); *** *p* < 0.001.

Psoriasis is a typical chronic inflammatory skin condition characterized by epidermal hypertrophy caused mostly by IL-17A [5,31]. Actually, IL-17A and TNF-α may operate together to activate the keratinocytes. IL-17A can stimulate the transcription of genes for several other inflammatory cytokines, such as IL-1β, IL-6, IL-8 and TNF-α [46]. Emerging research suggests that IL-1β plays a crucial role in psoriasis as well. By increasing IL-17 production in dermal γδ T-cell, the increased IL-1β might further exacerbate skin inflammation [30]. Considering the essential roles of the TNF-α/NF-κB/IL-17A axis in the pathogenesis of psoriasis and the considerable reduction of TNF-α-induced NF-κB activation and IL-17A production by GA in keratinocytes, we further assessed the therapeutic potential of GA in the OXA-induced psoriasis mouse model. The schematic diagram of the establishment and treatment procedure of this model is shown in Figure 4A. The repeated topical application of OXA resulted in increased ear thickness and weight and the appearance of erythema (Figure 4B,C). After topical GA treatment, the ear weight and thickness in the psoriasis-like mice were significantly decreased (Figure 4B,C). Histopathological examination of the ear tissue sections showed that the stimulation of OXA resulted in edema, inflammatory cell infiltration in the epidermis and dermis, as well as keratinocyte necrosis (Figure 4D). However, the symptoms were significantly relieved after GA smear treatment, which was also confirmed by the pathological score (Figure 4D,E). 

### 3.5. GA Ameliorate Skin Inflammation via Repressing the TNF-α/NF-κB/IL-17A Axis in ICD and Psoriasis-Like Mice

**Figure 5:**
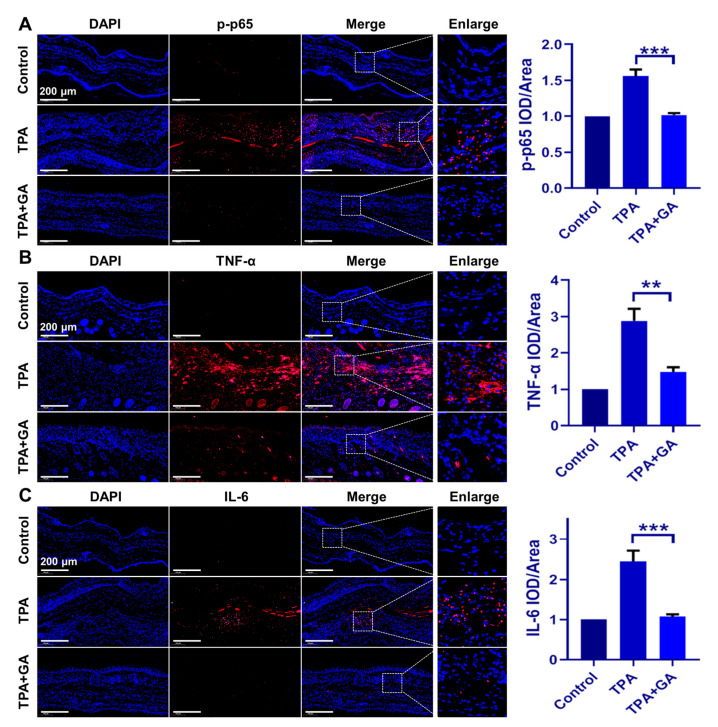
Immunofluorescence staining of mice ear samples following GA administration in the ICD model. Expression of p-p65, TNF-α and IL-6 is shown in red fluorescence, while the nucleus was stained in blue by DAPI. Experiments were undertaken to confirm the suppression of (**A**) p-p65, (**B**) TNF-α and (**C**) IL-6 expression by GA (50 μM), relative to the TPA-only group. The magnification for all images is the same. Magnification = 20×, Scale bar = 200 μm. The positive area in each group was qualitatively analyzed. The data are shown as mean ± SD of triple tests, ** *p* < 0.01, *** *p* < 0.001.

An ear tissue immunofluorescence assay was conducted to further elucidate the in vivo mechanism of GA treatment in ICD and psoriasis. In TPA-induced ICD mice, the immunofluorescence staining intensities of TNF-α, IL-6 and p-p65 were all increased in comparison to those in the control group (Figure 5A–C). After treatment with GA, their expression was significantly inhibited, indicating that AuNCs can suppress the overexpression of proinflammatory cytokines via blocking the activation of the TPA-induced NF-κB pathway in vivo, thereby ameliorating ICD symptoms. This is consistent with the in vitro results obtained for the keratinocytes.

**Figure 6:**
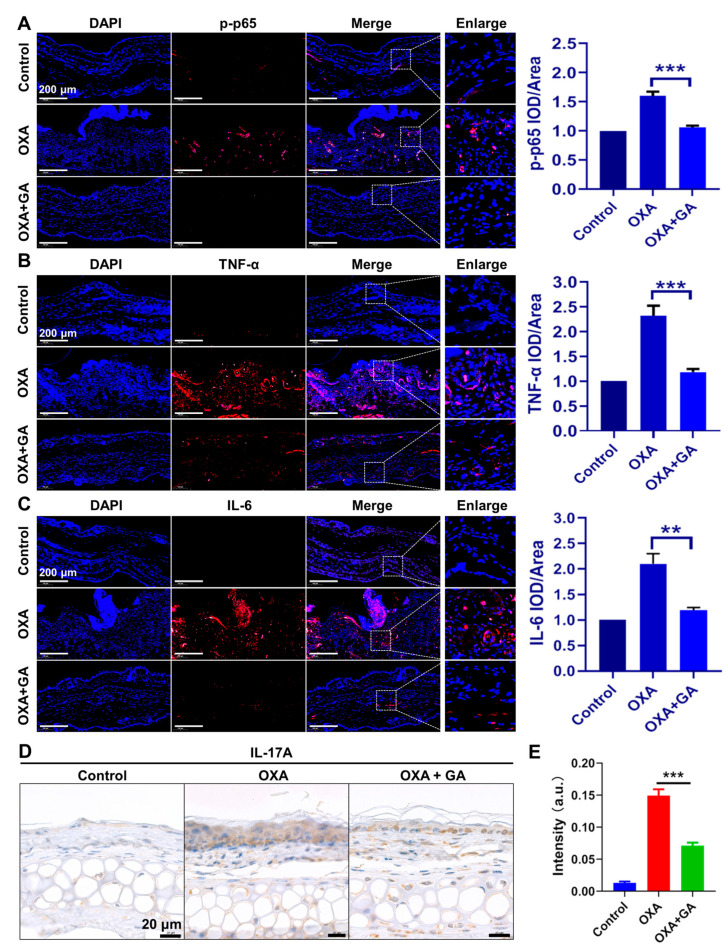
Effect of AuNCs on the TNF-α/NF-κB/IL-17A axis in psoriasis-like mice. In the OXA-induced psoriasis model, immunofluorescent detection of (**A**) p-p65, (**B**) TNF-α and (**C**) IL-6 is shown in red, whereas DAPI was used to stain the nucleus in blue. The magnification for all images is the same. Magnification = 20×, Scale bar = 200 μm. (**D**) OXA-stimulated mice ear sections were stained for IL-17A. Scale bar = 20 μm. (**E**) The intensity of IL-17A staining in each group was qualitatively analyzed. The data are presented as mean ± SD of triple tests, ** *p* < 0.01, *** *p* < 0.001.

In the OXA-induced psoriasis-like mice, the activation of p-p65 and the expression of TNF-α and IL-6 were also dramatically increased in the ear tissue but were effectively decreased after GA treatment (Figure 6A–C). IL-17A is a well-identified and the most crucial cytokine in psoriasis pathogenesis, whose transactivation in psoriatic keratinocytes is mediated by the TNF-α/NF-κB axis. Therefore, the expression of IL-17A in the ear tissue of the OXA-induced psoriasis-like mice was also qualitatively analyzed by immunohistochemistry. The results indicated that, compared with the OXA group, GA treatment significantly suppressed the transactivation of IL-17A in the ear tissues as well (Figure 6D,E). These data demonstrated that AuNCs could inhibit the expression of IL-17A through repressing the TNF-α/NF-κB axis in vivo and ameliorate psoriasis. 

### 3.6. The Topical Cutaneous Administration of GA Possesses High Biosafety

**Figure 7:**
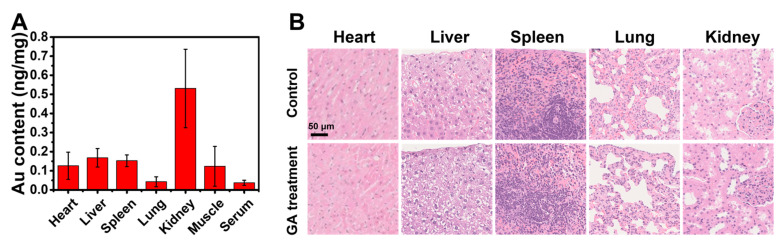
In vivo biodistribution of Au (**A**) and histopathological examination (scale bar = 50 µm, 40×) of major organs (**B**) after continuous topical treatment with GA (5 mg/kg Au) on the back of mice once a day for 7 days. The Au content was determined by ICP-MS, and the data are presented as mean ± SD of three mice.

The biodistribution of Au in vivo after continuous GA local treatment of the skin indicated that the topically administered AuNCs mainly accumulated in the kidneys after transdermal absorption and less in the liver and spleen, indicating that they might be excreted through the urinary system (Figure 7A). a histopathological examination of the major organs showed that the topical administration of AuNCs had no apparent effect on these tissues (Figure 7B). Overall, these results demonstrated that topically administered AuNCs possess high biocompatibility and biosafety in vivo.

## 4. Conclusions

In summary, this study demonstrated that gold nanoclusters (AuNCs) with peptides as ligands could significantly inhibit the NF-κB signal in TNF-α-treated keratinocytes and suppress the subsequent expression of proinflammatory cytokines mediated by the TNF-α/NF-κB axis, including IL-6, IL-1β and IL-17A, in vitro. Considering the crucial function of these cytokines in the pathogenesis of inflammatory skin diseases, especially the role of IL-17A in psoriasis, AuNCs were employed to test their therapeutic effect on inflammatory skin disease models. Interestingly, AuNCs significantly ameliorated TPA-induced acute irritant contact dermatitis and OXA-induced psoriasis-like symptoms in vivo. The suppression of the TNF-α/NF-κB/IL-17A axis in vivo by AuNCs was confirmed by immunostaining histopathologic sections. In addition, the AuNCs themselves did not cause any skin irritation or obvious abnormalities in the major organs, showing good biocompatibility and biosafety, which will facilitate their clinical application. Based on these results, the topical treatment with AuNCs may be a general effective therapy for inflammatory skin diseases. This direct therapeutic effect on immune-mediated skin diseases also opens a new avenue for the biomedical application of AuNCs.

## Data Availability

Data will be made available on request.

## Supplementary Materials

The following supporting information can be downloaded at: https://www.mdpi.com/article/10.3390/nano13040662/s1, Figure S1: TPA was applied topically to obtain a mouse ICD model, and cream was applied 45 min and 2 h after TPA stimulation.
