# Supplementary material for: Peptide-Protected Gold Nanoclusters Efficiently Ameliorate Acute Contact Dermatitis and Psoriasis via Repressing the TNF-α/NF-κB/IL-17A Axis in Keratinocytes
Source: Nanomaterials (Basel). 2023 Feb 8;13(4):662. doi: 10.3390/nano13040662 (PMC9963485; doi:10.3390/nano13040662)
Supplement: Supplementary file 1 [file nanomaterials-13-00662-s001.zip › nanomaterials-2177282-supplementary.pdf]

nanomaterials

MDPI

Supplementary information

Peptide-Protected Gold Nanoclusters Efficiently Ameliorate Acute Contact Dermatitis and Psoriasis via Repressing the TNF-α/NF-κB/IL-17A Axis in Keratinocytes

Yu Liu [1], Cong Meng [1], Yanggege Li [1], Dongfang Xia [2], Cao Lu [1], Jing Lai [1], Yulu Zhang [1], Kai Cao [1], Xueyun Gao [1,*] and Qing Yuan [1,*]

1 Center of Excellence for Environmental Safety and Biological Effects, Beijing Key Laboratory for Green Catalysis and Separation, Department of Chemistry, Beijing University of Technology, Beijing 100124, China; liuy111@emails.bjut.edu.cn (Y.L.); MengCong@emails.bjut.edu.cn (C.M.); liliygg@163.com (Y.L.); lucaobjut@163.com (C.L.); laijing0620@163.com (J.L.); lyz092800@163.com (Y.Z.); kcao@bjut.edu.cn (K.C.)

2 College of Chemistry and Material Science, Shandong Agricultural University, Taian 271018, China; xiadf@ihep.ac.cn

* Correspondence: gaoxy@ihep.ac.cn (X.G.); yuanqing@bjut.edu.cn (Q.Y.)

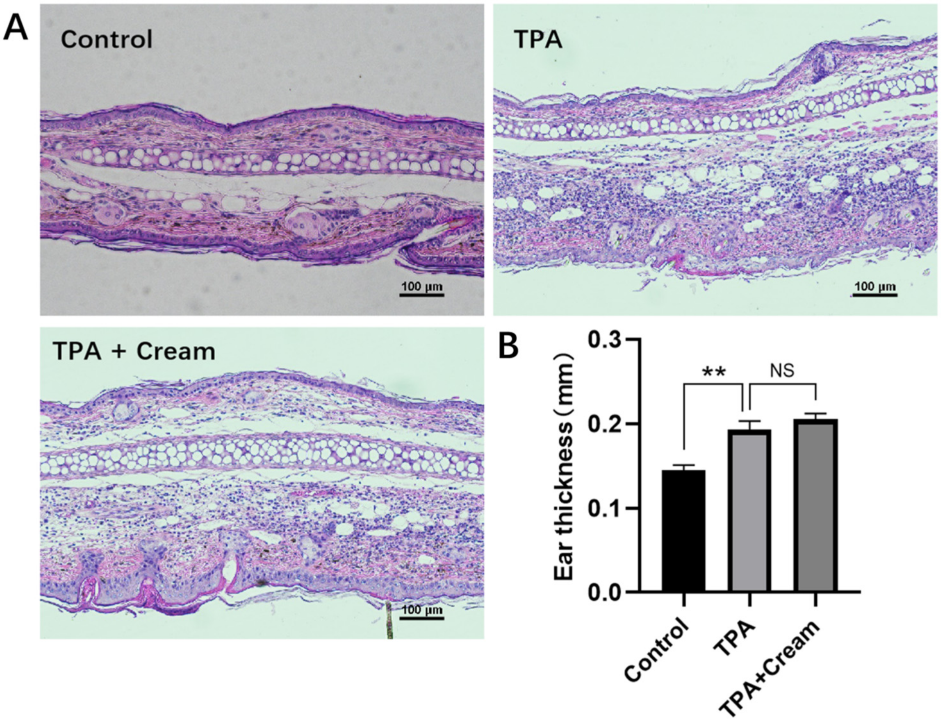

Figure S1. TPA was applied topically to induce a mouse ICD model, and cream was applied 45min and 2h after TPA stimulation. **(A)** Representative H&E-stained pathological sections of each group. The magnifications for all images are the same. Scale bar = 100 μm. Ear swelling was assessed by measuring ear thickness **(B)**. Data are presented as the mean±standard deviation (n=3); **p < 0.01, n.s., not significant.
